# GC1f Vitamin D Binding Protein Isoform as a Marker of Severity in Autism Spectrum Disorders

**DOI:** 10.3390/nu14235153

**Published:** 2022-12-03

**Authors:** Elisabetta Bolognesi, Franca Rosa Guerini, Stefano Sotgiu, Matteo Chiappedi, Alessandra Carta, Martina Maria Mensi, Cristina Agliardi, Milena Zanzottera, Mario Clerici

**Affiliations:** 1 IRCCS Fondazione Don Carlo Gnocchi ONLUS, 20148 Milan, Italy; 2 Unit of Child Neuropsychiatry, Department of Medicine, Surgery and Pharmacy, University of Sassari, 07100 Sassari, Italy; 3 Child Neuropsychiatry Unit, IRCCS Mondino Foundation, 27100 Pavia, Italy; 4 Department of Pathophysiology and Transplantation, University of Milan, 20122 Milan, Italy

**Keywords:** autism spectrum disorders (ASD), vitamin D binding protein (DBP), *GC* isoforms, pathogenesis, clinical severity

## Abstract

Autism spectrum disorders (ASD) are characterized by a wide spectrum of clinical, behavioral, and cognitive manifestations. It is, therefore, crucial to investigate possible biomarkers associated with specific ASD phenotypes. Ample literature suggests a possible role for vitamin D (VD) in influencing ASD clinical phenotypes. We analyzed three vitamin D binding protein gene (*DBP*) functional polymorphisms (rs2282679, rs7041, and rs4588), which are involved in the modulation of vitamin D serum concentration in 309 ASD children and 831 healthy controls. Frequency comparisons of single nucleotide polymorphisms (SNPs) alleles, genotypes, and *GC* isoforms (GC1f, G1s, and GC2)—generated by the combination of rs7041 and rs4588 alleles—were correlated with ASD diagnostic, behavioral, and functioning scales. The GC1f isoform was significantly more frequent in ASD compared with controls (18.6% vs. 14.5% pc = 0.02). Significantly higher scores for item 15 of the Childhood Autism Rating Scale (CARS) and lower ones for the Children’s Global Assessment Scale (CGAS) functioning scales were seen in ASD carrying the GC1f isoform. In *GC* phenotype analysis, a gradient of severity for overall CARS scores and CARS item 15 was observed, with scores decreasing according to the presence of GC1f-GC1f > GC1f-GC1s > GC1s-GC1s > GC1f-GC2 > GC2-GC2 isoforms. Similarly, lower CGAS scores were seen in carriers of the GC1f-GC1f isoform, whereas higher scores were present in those carrying GC2-GC2 (*p* = 0.028). This is the first study to evaluate possible relationships between *GC* variants and the different aspects of ASD in Italian ASD children. Results, although needing to be validated in ampler cohorts, suggest that the GC1f isoform could be a marker of severity in ASD that may be useful in establishing the intensity of therapeutic and rehabilitative protocols.

## 1. Introduction

Autism spectrum disorder (ASD) is a neurodevelopmental disorder that emerges in early infancy, resulting in different levels of impairment in communication and mutual social interaction associated with restricted, repetitive, and/or sensory behaviors and/or interests [1]. ASD prevalence has increased significantly in the last three decades, and according to the 2021 Italian National Institute of Health survey, 1 child in 77 is diagnosed with ASD [2]. The cause of ASD is still not known, although it is believed to result from a complex interaction of immunologic, environmental, genetic, and epigenetic factors [3,4,5]. Within this complex etiopathogenetic model, several reports suggest a pathogenic role for vitamin D, a molecule that is crucial in brain development, neuronal proliferation and differentiation, neurotransmission, and neuroplasticity [6,7,8,9]. Vitamin D is synthesized as cholecalciferol (25-OH-D) in the lower levels of the epidermis through a chemical reaction, which is dependent on sun exposure, specifically ultraviolet B (UVB) radiation. [10]. Maternal vitamin D deficiency during pregnancy may represent a risk factor for ASD in offspring [11]. Further, lower levels of vitamin D were reported in ASD children compared with neurotypical controls [12,13,14], and vitamin D supplementation was suggested to exert a potential beneficial effect in these children [15]. However, a clear consensus on the role of vitamin D in the pathogenesis of ASD has not been reached [16]. Different genes are involved in the vitamin D pathway, including (1) the vitamin D receptor (*VDR*) gene, a nuclear hormone receptor within the central nervous system [17]; (2) genes involved in the activation and degradation of vitamin D (*CYP2R1*, *CYP27B1*, and *CYP24A1*) [18]; (3) the vitamin D binding protein (*DBP*) gene, which codifies for the protein that binds vitamin D metabolites in plasma [19]. DBP has a unique binding site for all vitamin D metabolites, although affinity strength can vary, e.g., 25(OH) D affinity for DBP is 10-to-100-fold higher than that of 1.25 (OH)_2_ D. The DBP-25(OH)D complex forms a circulating reservoir of vitamin D, fighting hypovitaminosis D when the source of new vitamin D is impaired. DBP also regulates the entry of all vitamin D metabolites into tissues and cells [20].

DBP is coded by the *GC* gene on chromosome 4q11-q13, which includes several polymorphisms that influence serum vitamin D levels. The most important of these are the two functional polymorphisms rs7041 (c.1296A > C), encoding glutamic instead of aspartic acid at position 432 (p.Asp432Glu), and rs4588 (c.1307G > T), which encodes lysine instead of threonine at position 436 (p.Thr436Lys) [21]. These polymorphisms result in three major *GC* isoforms: (1) GC1s (rs7041C-rs4588G) coding for 432Glu/436Thr; (2) GC1f (rs7041A-rs4588G) coding for 432Asp/436Thr; and (3) GC2 (rs7041A-rs4588T) coding for 432Asp, 436Lys. The three isoforms modulate the concentration of circulating DBP and vitamin D [22,23] and generate six different phenotypes: GC1f-GC1f, GC1f-GC1s, GC1s-Gc1s, GC1f-GC2, GC1s-GC2, and GC2-GC2. Finally, the intronic rs2282679 SNP is similarly associated with the modulation of vitamin D concentration in physiology and pathology [24,25,26].

Recently, we showed a possible contribution of the FokI *VDR* polymorphism in ASD clinical heterogeneity; thus, the FokI (T) allele was strongly correlated with hyperactivity in children with ASD [27]. To further investigate the possible involvement of the vitamin D (VD) pathway in ASD, we genotyped a cohort of 309 ASD children and 831 healthy controls for the three polymorphisms of DBP (rs2282679, rs7041, and rs4588). We evaluated their distribution and the presence of possible correlations with ASD diagnostic, behavioral, and functioning scales. Results showed that GC isoforms, in particular GC1f, are indeed correlated with ASD clinical severity.

Treatment of ASD is still a debated topic, and even existing guidelines provide different and sometimes conflicting suggestions [28]. It is, therefore, useful to try to identify severity markers, which can be used to differentiate patients not only in terms of the severity of the clinical presentation (as it is done in Diagnostic and Statistical Manual of Mental Disorders, 5th Edition (DSM-5) [1]) but also in the intensity of the rehabilitative treatment to be offered. This, in turn, is highly relevant not only for the patient but could also be beneficial in terms of health management given the high costs of potentially effective non-pharmacological interventions [29].

## 2. Materials and Methods

### 2.1. Patients and Controls

Three hundred and nine (232 boys, 77 girls, mean age 8.2 ± 4.1 years) children with an ASD diagnosis according to the DSM-5 criteria [1] were enrolled at the IRCCS Mondino Foundation National Neurological Institute of Pavia (Italy) and the Child Neuropsychiatry Division, University of Sassari (Italy). One hundred and seventy-five children (134 boys, 41 girls, mean age 7.4 ± 4.08. years) were of Italian Peninsular descent, and the remaining 134 children were of Sardinian ancestry (98 boys, 36 girls, mean age 9.3 ± 3.8 years). All the sample cases were collected in past years in our repository; extensive neuropsychological and behavioral analyses were performed in a subgroup of 91 ASD children (71 boys, 20 girls, mean age 6.9 ± 3.7 years). The global cognitive status was evaluated by the Leiter Intelligence Scales [30], Wechsler Intelligence Scales [31], and Raven’s Progressive Matrixes [32]. Diagnostic tools that we used to measure the clinical symptom severity included the Autism Diagnostic Observation Schedule 2 (ADOS-2) [33], the semi-structured parent’s interview, Autism Diagnostic Interview-Revised (ADI-R) [34], and the Childhood Autism Rating Scale (CARS) [27,35]. The general functioning of children was assessed through the Children’s Global Assessment Scale (CGAS) [36], which provides a measure of the impact on global functioning for youths under the age of 18. Inclusion criteria were (a) age between 3 and 12 years, (b) a primary diagnosis of ASD, and (c) the results of the Autism Diagnostic Observation Schedule 2 (ADOS-2,33) test that measures the clinical symptoms of autism. Exclusion criteria were a diagnosis of psychotic disorders, intellectual disability, or other developmental disabilities according to DSM-5 criteria. Patients with an ascertained lesion of the central nervous system and/or a genetic syndrome were also ruled out from the study. Clinical assessment was blinded to the genotypes of the subjects.

The control group included 831 healthy Italian blood donors (331 men, 500 women, mean age 44.1 ± 12.5 years). Although no gender-related difference is reported for the frequency of the analyzed single nucleotide polymorphisms (SNPs), we preliminarily performed a regression analysis and confirmed the absence of a relation with sex. No age-matching was used since genetic data alone were used to compare cases and controls.

The study was designed and conducted according to the Declaration of Helsinki; the research protocol was approved by the Don Gnocchi Foundation Ethical Committee (06_18/05/2016).

### 2.2. Vitamin D Binding Protein SNPs Genotyping

For ASD subjects, venous blood in ethylenediaminetetraacetic acid (EDTA) or saliva was collected. Genomic DNA from blood was obtained using a standard phenol/chloroform procedure, whereas the ORAgene-DNA (DNA Genotek, Ottawa, ON, Canada) was used for saliva. The genomic DNA of healthy controls was extracted from venous blood in EDTA using a standard phenol/chloroform method.

The DBP SNPs rs2282679, rs7041, and rs4588 were estimated by real-time allelic discrimination using the TaqMan Assay probes (Applied Biosystems, Carlsbad, CA, USA) C___3129606_10, C___3133594_30, and C___8278879_10. Each reaction was performed in a 10 µL volume as follows: 1 µL of DNA/sample at the concentration of 10 ng/µL, 0.25 µL of 40X probe, 5.0 µL of TaqMan Genotyping Master Mix (Applied Biosystems, Carlsbad, CA, USA), and 3.75 µL of DNAse free water. Experiments were performed in 96-well plates, and amplification was performed on a CFX96^TM^ System (Bio-Rad, Hercules, CA, USA). Polymerase chain reaction (PCR) consisted of a hot start at 95 °C for 10 min followed by 40 cycles at 94 °C for 15 s and 60 °C for 1 min. Fluorescence detection took place at 60 °C. In each experiment, control samples of known genotypes and a negative control were included. After amplification, an allelic discrimination plot was generated by the software, showing homozygote clusters, heterozygote clusters, and the negative controls, allowing genotyping of the samples.

### 2.3. Statistical Analysis

To detect possible genotyping errors, we measured the Hardy–Weinberg equilibrium (HWE) for the rs2282679, rs7041, and rs4588 SNPs polymorphisms in both cases and controls. The analysis was performed using a Chi-square method. 2 × 2 contingency tables were used to compare the distribution of rs2282679, rs7041, and rs4588 alleles in cases vs. controls. Genotype and haplotype distribution between groups were compared using 2 × N contingency tables. When a statistically significant result was found, a 2 × 2 contingency table was applied, and the resulting *p*-value was corrected for the degree of freedom (DF). The odds ratio (OR) and its 95% confidence interval (CI) were used to measure the association of each polymorphism/genotype with the disease. The *p*-value was considered significant at <0.05 after Bonferroni correction for the proper degrees of freedom (p_c_). The non-parametric Kolmogorov–Smirnov test was used to verify the normal distribution of the scores of clinical, behavioral, and functioning scales in ASD patients. Since most of the clinical, behavioral, and functioning scales did not fit a normal distribution, non-parametric Kruskal–Wallis and Mann–Whitney tests were used to measure the association with DBP polymorphisms/haplotypes with scores of the examined scales. When a non-parametric test resulted in statistical significance, a post hoc pairwise comparison was performed to determine which isoform/haplotype significantly differed from one another.

A univariate analysis (logistic regression) was applied to confirm no relation with sex in the distribution of the studied SNPs. Data were analyzed by SPSS version 28.0 (IBM Corp. in Armonk, NY, USA) and the open source openEpi https://www.openepi.com (accessed on 1 October 2022).

## 3. Results

### 3.1. DBP rs2282679, rs7041, and rs4588 Genotype Distribution in ASD Children and Healthy Controls

The allele and genotype distribution of *DBP* rs2282679, rs7041, and rs4588 in ASD children and in healthy controls are presented in Table 1. Genotype frequency for each polymorphism was in the Hardy–Weinberg equilibrium both in ASD and healthy controls. In healthy controls, the frequency of the rs2282679, rs7041, and rs4588 minor alleles agreed with previously reported results [37,38]. rs2282679, rs7041, and rs4588 allelic and genotypic distribution was then compared with ASD children from Sardinia and continental Italy; since no differences were found, the two groups were combined. No difference in allelic and genotype frequencies was observed when ASD children were compared with healthy controls (Table 1). Finally, no differences in SNPs frequencies were found when both ASD children and healthy controls were stratified according to their gender (not shown).

### 3.2. GC Isoform and Phenotype Distribution in ASD Children and Healthy Controls

Analyses of the distribution of the GC isoforms, obtained by the combination of rs7041 (A/C) with rs4588 (A/G) alleles, namely GC1s (C/G), GC1f (A/G), and GC2 (A/T), showed the presence of significant differences in their distribution in ASD children compared with healthy controls (*p* = 0.04). Specifically, the GC1f (A/G) combination was significantly more frequent in ASD children (18.6%) than in healthy controls (14.5%) (*p* = 0.01, pc = 0.02, OR: 1.37, 95% CI: 1.07–1.74). Finally, no difference in isoform phenotype distribution was seen between ASD children and healthy controls (Table 2).

### 3.3. GC Isoform Genotype and Phenotype Correlation with Clinical Symptoms Severity, Cognitive, Behavioral, and Functioning Scales

**Figure 1:**
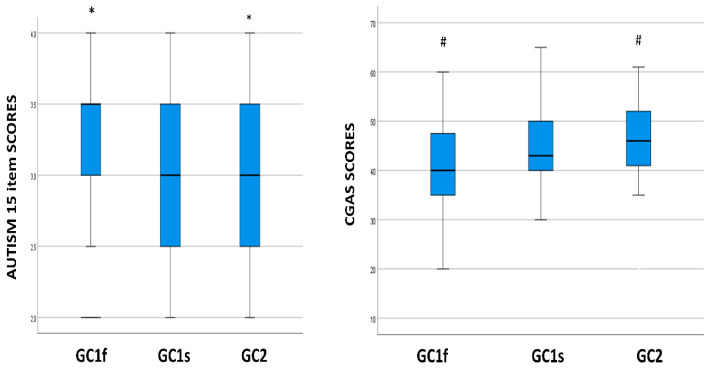
Boxplot of the distribution of autism 15 item and CGAS scores in relationship with the *GC* isoform genotypes. Within each box black lines denote median values. Statistically significant pairwise comparisons are reported: *: *p* = 0.014 and #: *p* = 0.004. CGAS: Children’s Global Assessment Scale.

Genetic and phenotype correlations of the GC isoforms with results from Leiter’s and Wechsler Intelligence Scales, and CARS, ADOS-2, ADI-R, and CGAS tests were evaluated in a subgroup of 91 ASD children to analyze the possible impact of these genetic parameters on clinical severity symptoms, cognitive, behavioral, and functional ASD parameters. Kruskal–Wallis analyses showed that GC isoforms were significantly correlated with item 15 (i.e., autism general impression) of the CARS scale (*p* = 0.013), as well as with CGAS scores (which assess the general adaptive functioning of children) (*p* = 0.005). Specifically, the GC1f isoform was associated with higher scores on CARS item 15 (median value: 3.3; interquartile range (IQR): 0.5) and lower scores on the CGAS scale (median value: 40.0; IQR: 12.3), whereas GC2 was associated with lower scores on CARS item 15 (median value 3.0; IQR: 1.0) (*p* = 0.014) and higher scores on the CGAS scale (median value: 46.0; IQR: 11) (*p* = 0.004) (Figure 1). No association of *GC* genotype distribution was observed with the other scales.

### 3.4. GC Isoform Phenotype Correlation with CARS Total Scores

**Figure 2:**
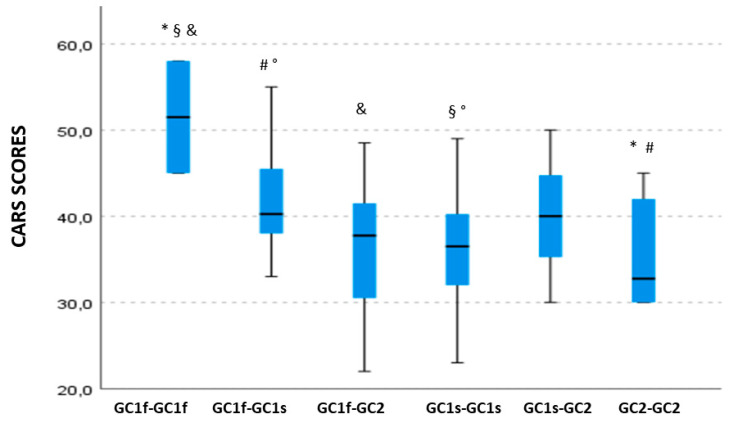
Boxplot of the distribution of CARS scores in relationship with the GC isoform phenotypes. Within each box black lines denote median values. Statistically significant pairwise comparisons are reported: *: *p* = 0.021 §: *p* = 0.024 &: *p*= 0.035 °: *p* = 0.018 and #: *p* = 0.049.

A significant association was observed between GC isoforms and CARS total score (*p* = 0.032). In detail, a decreasing gradient could be observed for higher median CARS scores, as follows: GC1f-GC1f > GC1f-GC1s > GC1s-GC1s > GC1f-GC2 > GC2-GC2; notably, CARS score severity decreased if GC1f was replaced by GC2 (Figure 2). Post hoc pairwise comparison showed that CARS scores of ASD children carrying the GC1f-GC1f isoforms were significantly higher (median value: 51.5; IQR: 6.5) compared with ASD children carrying GC1f-GC2 (median value: 37.8; IQR. 9.5; *p* = 0.035), GC1s-GC1s (median value: 36.5; IQR: 8.13; *p* = 0.024), or the GC2-GC2 isoforms (median value: 32.8; IQR: 9.38; *p* = 0.021). Finally, significantly higher CARS scores were observed in children carrying GC1f-GC1s (median value: 40.3; IQR: 7.13) compared with those carrying GC1s-GC1s or GC2-GC2 isoforms (*p* = 0.018 and *p* = 0.049, respectively).

### 3.5. GC Isoform Phenotype Correlation with Item 15 of the CARS Scale Scores

**Figure 3:**
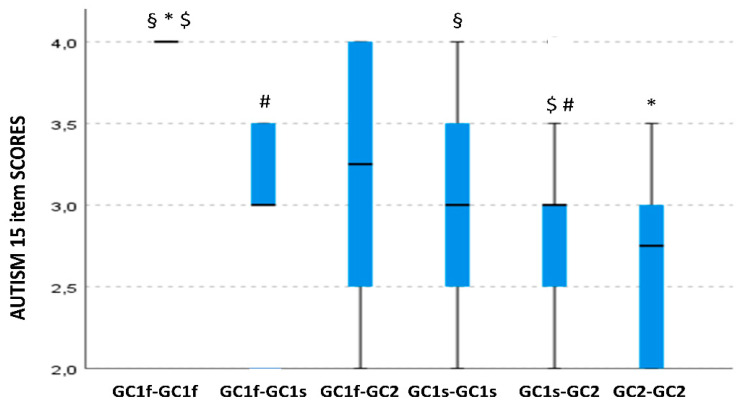
Boxplot of the distribution of autism 15 item scores in relationship with the GC isoform phenotypes. Within each box black lines denote median values. Statistically significant pairwise comparisons are reported: *: *p* = 0.008 §: *p* = 0.02 $: *p*= 0.01 and #: *p*= 0.049.

GC isoform phenotype was also significantly associated with scores on item 15 of the CARS scale (*p* = 0.038). Pairwise comparisons showed the same trend when correlations with CARS total scores were analyzed. Thus, higher scores were seen in GC1f-GC1f (median value: 4.0; IQR: 0.1) compared with either GC2-GC2 (median value: 2.75; IQR: 0.87; *p* = 0.008) or GC1s-GC1s ASD children (median value; 3.0; IQR: 1.0; *p* = 0.020). Furthermore, higher scores were detected in GC1f-GC1f (median value: 4.0; IQR: 0.1) compared with GC1f-GC1s (median value: 3.0; IQR: 0.5; *p* = 0.01) and GC1s-GC2 ASD children (median value 3.0; IQR: 0.5; *p* = 0.049). Also, in this case, a gradient of severity was noticed when GC1f was present in homozygosis as follows: GC1f-GC1f > GC1f-GC1s > GC1s-GC1s > GC1s-GC2 > GC2-GC2 (Figure 3). 

### 3.6. GC Isoform Phenotype Correlation with CGAS Scores

**Figure 4:**
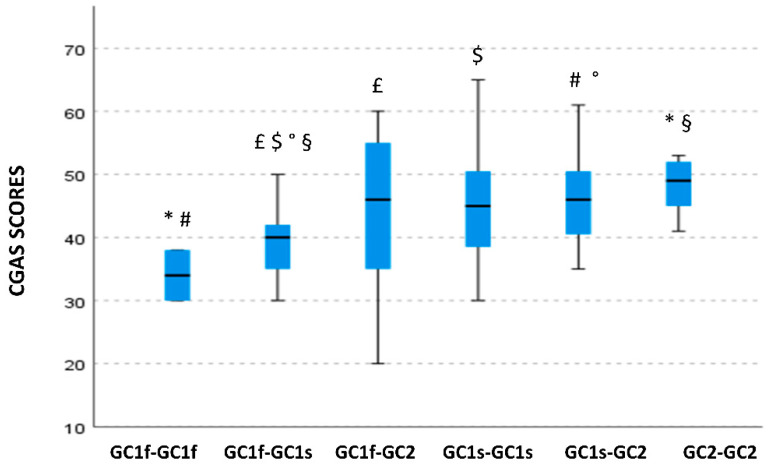
Boxplot of the distribution of CGAS scores in relationship with the GC isoform phenotypes. Within each box black lines denote median values. Statistically significant pairwise comparisons are reported: *: *p* = 0.028 #: *p* = 0.049 £: *p* = 0.048 $: *p* = 0.019 °: *p* = 0.007 and §: *p* = 0.012.

A significant skewing of CGAS scores among different GC isoform phenotypes could be observed as well (*p* = 0.016). Specifically, children carrying the GC1f-GC1f isoform phenotype were characterized by lower (more severe) CGAS scores (median value: 34.0; IQR: 4.0) compared with those carrying the GC2-GC2 isoform phenotype (median value: 49.0; IQR: 6.75; *p* = 0.028). Additionally, the GC1f-GC1f phenotype was associated with lower CGAS scores compared with the GC1s-GC2 (median value: 46.0; IQR: 10; *p* = 0.049). CGAS scores were also significantly reduced in GC1f-GC1s carriers (median value: 40.0; IQR: 6.75) compared with either GC1f-GC2 (median value; 46; IQR: 19.8), GC1s-GC1s (median value: 45; IQR: 11), GCs-GC2 (median value: 46.0; IQR: 10), or GC2-GC2 carriers (median value: 49.0; IQR: 6.75;), *p* = 0.048, *p* = 0.019, *p* = 0.007 and *p* = 0.012, respectively (Figure 4).

Notably, no significant associations were detected between any GC isoform phenotypes and Leiter’s and Wechsler Intelligence Scales, ADOS-2, or ADI-R scores (data not shown).

### 3.7. Lack of Correlations between DBP rs2282679 and Clinical, Behavioral, and Functioning Scales

Finally, and in contrast with the above results, no correlation could be found between DBP rs2282679 alleles and/or genotypes and any of the clinical, behavioral, and functioning scales (data not shown).

## 4. Discussion

Vitamin D is suspected of playing a role in the pathogenesis of ASD, although an unequivocal agreement on this issue has not been reached. This is, at least, partly due to the great complexity of the vitamin D pathway, which includes several different proteins, amongst which the vitamin D binding protein (DBP) plays a pivotal role. DBP is encoded by the *GC* gene, which includes several different polymorphisms, and binds different vitamin D metabolites in blood, moving them between the skin, liver, kidney, and target tissues.

We verified whether *GC* polymorphisms were differently distributed in ASD children and, if that was the case, if they correlated with parameters of clinical severity. Results showed that the GC1f (rs7041A-rs4588G) genotype was significantly more likely to be observed in children with ASD. Notably, this polymorphism was significantly correlated with higher scores on item 15 of the CARS scale—which represents the global clinical severity of ASD symptoms according to clinical observation—and with lower scores on the CGAS functioning scale. Moreover, association analyses showed a gradient of severity between *GC* polymorphisms, total CARS scores, and item 15 CARS scores, with higher scores (increased clinical severity) seen in GC1f-GC1f carriers and lower scores in GC2-GC2 carriers. Similarly, ASD children carrying GC1f-GC1f had lower CGAS scores, indicating a worse general adaptive functioning behavior than those carrying GC2-GC2. Overall, the presence of the GC1f isoform in homozygosis or heterozygosis was seen to be associated with more severe ASD clinical manifestations and worse general functioning.

DBP transports around 85–90% of circulating vitamin D metabolites and is synthesized by one of the most polymorphic genes in humans. The distribution of the different GC isoforms varies among populations living in different geographical areas [22,39], and these isoforms are suggested to be associated with diverse affinities for vitamin D [40,41,42,43,44,45]. Results obtained in a case–parent triad by means of log-linear and ETDT (Extended Transmission Disequilibrium Test) analyses indicated that the DBP rs4588 genotype is associated with ASD [46]. This is the only study linking DBP common gene variants to ASD. Other results showed that the plasma concentration of DBP is significantly reduced in ASD children [47]. The plasma concentration of vitamin D itself was reported to be reduced in these children [13,14,15], with a negative correlation between circulating serum vitamin D concentration and CARS scores, suggesting ASD severity to be associated with vitamin D serum levels [48,49].

Of note, DBP could play a role in ASD pathogenesis in more than one way. Thus, DBP is characterized by important immunoregulatory properties. Recent results indicate that only 2% of DBP functions as a vitamin binder, while its main effect is to modulate inflammation [50]. DBP is detectable in serum and cerebrospinal fluid [51], and it functions as a precursor of GcMAF, a protein driving macrophage activation, switching them into a proinflammatory phenotype. DBP transformation into GcMAF is promoted by B and T lymphocytes and is mediated by a cascade of carbohydrate processing reactions [52,53]. GC isoforms generate DBP proteins with different abilities to be converted into GcMAF because of their different degrees of glycosylation: GC1f and GC1s are transformed in GcMAF, but less than 10% of the GC2 isoforms are glycosylated and generate GcMAF. Therefore, inflammation is reduced in GC2 compared with GC1 phenotypes [54]. Supporting this finding are results showing that the GC1f genotype is associated with an augmented risk of chronic inflammatory diseases. Thus, (1) in chronic obstructive pulmonary disease (COPD), GCf1 was strongly associated with the risk of disease [55]; (2) in bronchiectasis, patients carrying the GC1f isoform have a more severe disease and more chronic infections in comparison with those without the GC1f isoform [56]; (3) in inflammatory bowel disease the GC2 isoform was less frequently observed in patients compared with healthy controls, suggesting a protective role [57]. On the other hand, the same GC2 isoform was associated with asthma susceptibility in the Chinese Han population [58].

The work of Schmidt [46] is the only one investigating the role of *GC* polymorphism in ASD. Results showed that the risk for ASD was increased in children inheriting from fathers the AA genotype of the rs4581 *GC* gene (vitamin D binding protein). Recently, an association between serum human endogenous retrovirus (HERV)-W-specific antibodies (Abs) and global adaptive functioning in ASD children was described [59]. These results, besides suggesting a possible use of such Abs to monitor clinical severity in ASD, reinforce the suggestion that immune activation and chronic neuro-inflammation are present in ASD [60,61,62]. To summarize: (1) autoptic evidence of abnormally activated microglia and astrocytes is described in ASD [63,64]; (2) increased concentration of proinflammatory cytokines, including interleukin-6, tumor necrosis factor-alpha, and interferon-gamma, are observed in ASD [65,66,67,68]; and (3) multiple inflammasome complexes are abnormally activated in ASD children [63,69,70,71,72,73,74,75]. The observation that non-GC2 polymorphisms are associated with increased macrophage activity and a higher degree of inflammation [76,77] allows the speculation that this genetic profile supports the worst clinical parameters seen in ASD GC1f-GC1f carriers. This possibility, though, is at least in part contradicted by the realization that the same non-GC2 polymorphisms correlate with a higher concentration of 1.25 (OH)_2_D_3_ whose binding to its intracellular receptor (VDR) results in the down-regulation of inflammation [78,79,80,81].

These speculations notwithstanding, results herein suggest that the GC1f isoform is associated with increased severity in ASD. The limitations of this study are the missing vitamin D and DBP plasma concentrations and the relatively small panel of ASD patients used for the correlation analysis between clinical parameters and DBP variants. Further investigation in a larger cohort of ASD children and a more in-depth evaluation of DBP expression in relationship with clinical assessment is required. In addition, we cannot exclude that a highly polymorphic gene, such as DBP with over 120 variants, or other SNPs could be more relevant to the disease outcome.

## 5. Conclusions

The vitamin D binding protein, GC1f isoform, is significantly more frequent in ASD children than in healthy controls. GC1f and GC1f-GC1f correlate with more severe ASD clinical manifestations (higher CARS scores) and worse general functioning (lower CGAS scores). The GC1f isoform is associated with increased severity in ASD and may be a useful genetic marker to plan the quality and intensity of rehabilitative protocols.

## Figures and Tables

**Table 1:** Allele and genotype distributions of rs2282679, rs7041, and rs4588 SNPs in ASD children and healthy controls (HC).

Allele Frequency	Continental ASD N (%)	Sardinian ASD N (%)	*p* Value	ASD N (%)	HC N (%)	*p* Value
rs2282679						
T	265 (75.7)	198 (73.8)		463 (74.9)	1223 (73.6)	
G	85 (24.3)	70 (26.2)	0.6	155 (25.1)	439 (26.4)	0.5
rs7041						
A	157 (44.8)	111 (41.4)		268 (43.4)	680 (40.9)	
C	193 (55.2)	157 (58.2)	0.4	350 (56.6)	982 (59.1)	0.3
rs4588						
T	83 (23.7)	70 (26.1)		153 (26.1)	439 (26.4)	
G	267 (76.3)	198 (73.9)	0.5	465 (75.3)	1223 (73.6)	0.42
Total	350	268		618	1662	
Genotype frequency						
rs2282679						
TT	101 (57.7)	72 (57.7)		173 (56.0)	447 (53.8)	
GT	63 (36.0)	54 (40.3)		117 (37.9)	329 (39.6)	
GG	11 (6.3)	8 (6.0)	0.7	19 (6.1)	55 (6.6)	0.8
rs7041						
AA	37 (21.1)	23 (17.2)		60 (19.4)	134 (16.1)	
AC	83 (47.4)	65 (48.5)		148 (47.9)	412 (49.6)	
CC	55 (31.4)	46 (34.3)	0.6	101 (32.7)	285 (34.3)	0.4
rs4588						
TT	10 (5.7)	8 (6.0)		18 (5.8)	54 (6.5)	
GT	63 (36.0)	54 (40.3)		117 (37.9)	331 (39.8)	
GG	102 (58.3)	72 (53.7)	0.7	174 (56.3)	446 (53.7)	0.7
Total	175	134		309	831	

N: absolute number of alleles/genotypes, %: allele/genotype frequency, ASD: autism spectrum disorders.

**Table 2:** GC isoform genotype and phenotype distribution in ASD children and healthy controls (HC).

Isoform	Genotype (rs7041/rs4588)	ASD N (%)	HC N (%)	*p*	OR, 95% IC
GC1s	C/G	350 (56.6)	1002 (60.3)		
GC1f	A/G	115 (18.6)	241 (14.5)	0.01, p_c_ = 0.02	1.367, (1.07–1.74)
GC2	A/T	153 (24.8)	439 (26.4)		
				0.04	
Isoform phenotype					
GC1f-GC1f	A/G-A/G	8 (2.6)	16 (1.9)		
GC1f-GC1s	A/G-C/G	65(21.0)	145 (17.4)		
GC1s-GC1s	C/G-C/G	101 (32.7)	285 (34.3)		
GC1f-GC2	A/G-A/T	34(11.0)	64 (7.7)		
GC1s-GC2	C/G-A/T	83(26.9)	267 (32.1)		
GC2-GC2	A/T-A/T	18 (5.8)	54 (6.5)		
				0.2	

N: absolute number of genotype/isoform phenotype, %: genotype/isoform phenotype frequency, *p*: uncorrected *p* value, p_c_: *p* value after Bonferroni’s correction for degree of freedom (DF), OR: odds ratio, 95% IC: interval of confidence.

## Data Availability

The data presented in this study are available upon request from the corresponding author.
